# Association of oral lichen planus with hepatic disorders and hepatocellular carcinoma: systematic review and meta-analysis

**DOI:** 10.4317/medoral.25661

**Published:** 2023-02-18

**Authors:** Miguel Ángel González-Moles, Teresa de Porras-Carrique, Pablo Ramos-García

**Affiliations:** 1 School of Dentistry, University of Granada. Biohealth Research Institute (Ibs.Granada) Granada, Spain

## Abstract

**Background:**

Oral lichen planus (OLP) is a prevalent autoimmune chronic inflammatory disease of unknown etiology. The importance of the association between hepatic disease and OLP lies in the fact that many of these disorders (HC, HB, cirrhosis, hepatic steatosis) behave as risk factors for hepatocellular carcinoma.

**Material and Methods:**

We searched PubMed, Embase, Web of Science, and Scopus for studies published before January 2022. We evaluated the quality of studies (Joanna Briggs Institute tool). We performed meta-analyses, investigated the heterogeneity between studies, and we also carried out subgroups, meta-regression, and small-study effects analyses. 146 studies (21,187 patients) were included in this study. Our study aims to evaluate current evidence on the prevalence and magnitude of association between hepatic diseases (especially those with risk of malignancy), hepatocellular carcinoma and OLP.

**Results:**

Our results suggest that patients with OLP present a significant tendency to the development of hepatitis B (OR=1.62, 95%CI=1.01-2.40, *p*=0.02), hepatitis C (OR=4.09, 95%CI=2.77-6.03, *p*<0.001), cirrhosis (OR=5.58, 95%CI=1.83-16.96, *p*=0.002), hepatic steatosis (OR=5.71, 95%CI=0.97-33.60, *p*=0.05) and hepatocellular carcinoma (OR=3.10,95%CI=1.14-8.43, *p*=0.03).

**Conclusions:**

Patients with OLP should be investigated to rule out the presence of hepatic disease, which can lead to hepatocellular carcinoma, allowing an early diagnosis that would help to a better approach to liver disease and a noTable improvement in prognosis in terms of both progression and severity.

** Key words:**Oral lichen planus, hepatocellular carcinoma, hepatitis, systematic review, meta-analysis.

## Introduction

Oral lichen planus (OLP) is a relapsing chronic disease, of unknown etiology, whose pathogenesis underlies a T-lymphocyte-mediated autoimmune aggression against the epithelial cells of the oral mucosa (1). OLP is a prevalent disease affecting 1.32% of the general population in Europe, with a significant and progressive increase in the prevalence reported from the age of 40 years onwards (2). Moreover, OLP is frequently associated with other pathologies, including emotional disorders (depression and anxiety), which affect more than 30% of patients (3) and autoimmune diseases (i.e., thyroid disease, type 1 diabetes, rheumatoid arthritis, fibromyalgia) (4); In addition, primary-level studies have reported an association between OLP and hepatic diseases, essentially hepatitis C and B (HC and HB respectively), liver cirrhosis, non-alcoholic hepatic steatosis, etc. (5-7). The reasons for this association are unknown, although in relation to HC there are highly suggestive findings indicating that HCV RNA is present in oral epithelial cells in 100% of patients with HC who develop OLP, while it does not appear in any patients with OLP without HC (8). El Tawdy and Rashed (9) also have hypothesized that HCV induces modifications in infected oral mucosal cells that could act as antigens resulting in a cytotoxic T lymphocyte response or are responsible for the initiation of a humoral response followed by the production of antibodies against virus-modified host cellular components. The importance of the association between hepatic disease and OLP lies in the fact that many of these disorders (HC, HB, cirrhosis, hepatic steatosis) behave as risk factors for hepatocellular carcinoma (10). However, to date, no high-level evidence-based studies have been published addressing the possible association between hepatic diseases predisposing to hepatocellular carcinoma and OLP, and no such study has been published analyzing the risk of developing hepatocellular carcinoma in patients with OLP.

Based on this background, we have decided to carry out a systematic review and meta-analysis to analyze the prevalence and magnitude of association between hepatic diseases (especially those with risk of malignancy), hepatocellular carcinoma and OLP.

## Material and Methods

This systematic review and meta-analysis closely followed the criteria of Cochrane Handbook for Systematic Reviews of Interventions and Joanna Briggs Institute (University of Adelaide, Australia) for systematic reviews formulating focused questions of prevalence and for proportion meta-analyses. It was also designed, conducted and validated according to AMSTAR2 high standards (11), and reporting complied with MOOSE and PRISMA guidelines.

- Protocol

A protocol was designed and submitted to PROSPERO International prospective register of systematic reviews (https://www.crd.york.ac.uk/PROSPERO; ID 311154) in order to enhance the transparency, precision, and integrity of our study. Our protocol meets updated PRISMA-P guidelines to guarantee a thorough approach (12).

- Search strategy

We searched PubMed, Embase, Web of Science, and Scopus databases for studies published before January 2022, with no publication date or language restraint. The search was carried out by combining free terms and thesaurus in all databases, using the keyword “oral lichen planus”, with the aim of maximizing sensitivity and finding a greater number of published articles related to OLP (Supplement 1, 2). We also included studies found by hand searching methods. All references were managed with Mendeley v.1.19.8 (Elsevier, Amsterdam, The Netherlands).

- Eligibility criteria

Inclusion criteria: 1. Original studies, with no publication language or year restrictions; 2. Observational study design; 3. Studies analyzing the prevalence of hepatic diseases in patients with OLP (not being strictly necessary the presence of a control sample), and/or the magnitude of association (with control group); 4. When data derived from the same sample of patients, it was selected depending on the amount of data provided and year of publication. Name and membership of authors, location of the study, recruitment period and source of patients were scrupulously contrasted to differentiate populations in studies.

Exclusion criteria: 1. Genital or cutaneous lichen planus with no oral lesions; 2. Lack of essential data for meta-analyses; 3. Reviews or meta-analyses, meeting abstracts, editorials, book chapters, retractions, letters, case reports or personal comments; 4. Studies performed in animals or *in vitro*; 5. Lack of healthy control group to study the magnitude of association.

- Study selection process

Eligibility criteria were applied independently by two authors (TDPC and PRG). Evaluators were first calibrated for the process of identification and selection of studies, performing several training rounds (50 papers each). Articles were selected in two stages, first, screening titles and abstracts for those apparently meeting inclusion criteria; second, reading the full-text of previously selected articles, excluding those not meeting eligibility criteria. The reliability of the study selection process was estimated calculating and inter-agreement score and a Cohen’s kappa (κ) value (99.53% of agreement; κ = 0.91).

- Data extraction

Data extraction was performed jointly with a third senior author, who had a role supporting the extraction of confusing datasets not clearly reported by primary-level studies. Datasets were managed using Excel v.16.53 spreadsheets (Microsoft. Redmond, WA, USA) to collect the following information: first, corresponding and last author, sample size, location of the study (nation and mainland), publication language (non-English language studies were translated using Google Translator), recruitment and follow-up periods, publication year, source of patient, recruiting, study design, diagnostic criteria for OLP and hepatic disorders (included in the Supplement 3), frequencies of hepatic diseases in patients with OLP, type of hepatic diseases (if were specified on the studies), clinical manifestations and site of OLP lesions, mean age, percentage of females, and tobacco and/or alcohol consumption.

- Evaluation of quality and risk of bias

A tool for systematic reviews (Joanna Briggs Institute, University of Adelaide, Australia) was used by two authors (TDPC and PRG) evaluating the RoB and quality addressing prevalence questions, specific for meta-analyses dealing with proportions.

- Statistical analysis

We calculated the prevalence of hepatic diseases in patients with OLP by the extraction of raw numerators (number of patients with hepatic diseases) and denominators (number of patients with OLP). Hence, we obtained proportions expressed as percentages accompanied by their corresponding 95% confidence intervals (95% CI). Freeman-Tukey double-arcsine transformation was used to minimize the influence of studies with extreme values (0, 100, or close to 0 or 100) and to stabilize the variance of the study-specific prevalence. The magnitude of association between OLP and hepatic diseases was also calculated combining odds ratios (OR) with 95% CI. Random effect models, following the method described by DerSimonian and Laird, were applied to carry out all meta-analyses in order to explain the differences between the study subgroups. We also constructed forest plot to represent the global results, considering *p*<0.05 as significant (Supplement 4-38).

Inter-study heterogeneity was evaluated using the χ-based Cochran’s Q test (*p*<0.10 was considered significant due to its poor statistical competence). We also assessed I2 statistic (interval values across 50-75-100% reflected a moderate-high degree of variance across the studies) to estimate what proportion of the variance in observed effects reflects variation in true effects, rather than sampling error (13,14).

Stratified meta-analyses were designed in advance with the purpose of assessing the pooled proportions of the different study subgroups on the prevalence of hepatic diseases in patients with OLP, as well as finding possible causes of heterogeneity. Furthermore, we carried out meta-regression analyses applying the restricted maximum likelihood (REML) method, and Monte Carlo simulations (10,000 permutations per meta-regression) to calculate the *p* values because of the lack of studies available studying each covariate (Supplement 4).

Secondary analyses were finally carried out to measure the stability and reliability of our results. We constructed funnel plots (Supplement 39-45) and used the original Egger regression test to try to confirm the absence of small-study effects, performing a linear regression of the effect estimates (i.e., transformed proportions) on their standard errors, weighting by 1/(variance of the effect estimate), considering a pEgger-value <0.10 as significant. PRG designed the statistical analysis and TDPC performed it operating with Stata software (version 16.1, Stata Corp, USA).

- Validation of methodological quality

Our systematic review and meta-analysis was acutely assessed by two authors (PRG and TDPC) using AMSTAR2 (15). Thus, 16 items (Supplement 46) were critically evaluated with this tool, obtaining an overall score, which was classified as “Critically low”, “Low”, “Moderate”, or “High”, depending on on the lack of strengths in critical (i.e., items: 2, 4, 7, 9, 11, 13, and 15) and non-critical domains. The full explanation can be found in the Supplement 47.

## Results

- Results of the literature search

The process of selecting potentially eligible studies is represented by the flow diagram (Supplement 2). Before January 2022, we retrieved 13,782 studies from four different databases (3,680 from PubMed, 3,487 from Embase, 3,449 from Scopus, and 3,166 from Web of Science, Supplement 1) and 17 from handsearching methods. Duplicates were removed and 5,120 titles and abstracts were screened next. After the exclusion of 3,339 studies in this phase, 1,781 were read full-text and, of these, only 146 met the inclusion criteria and were eligible (Supplement 48). The excluded studies in the second phase are listed in the Supplement 49.

- Study characteristics

Characteristics of the 146 included studies are summarized in Table 1. In the Supplement 3, the Table shows more descriptive characteristics of these studies, for example, the criteria applied for the diagnosis of OLP (e.g., WHO 1978, or van der Meij and van der Waal 2003).

- Qualitative evaluation

**Figure 1:**
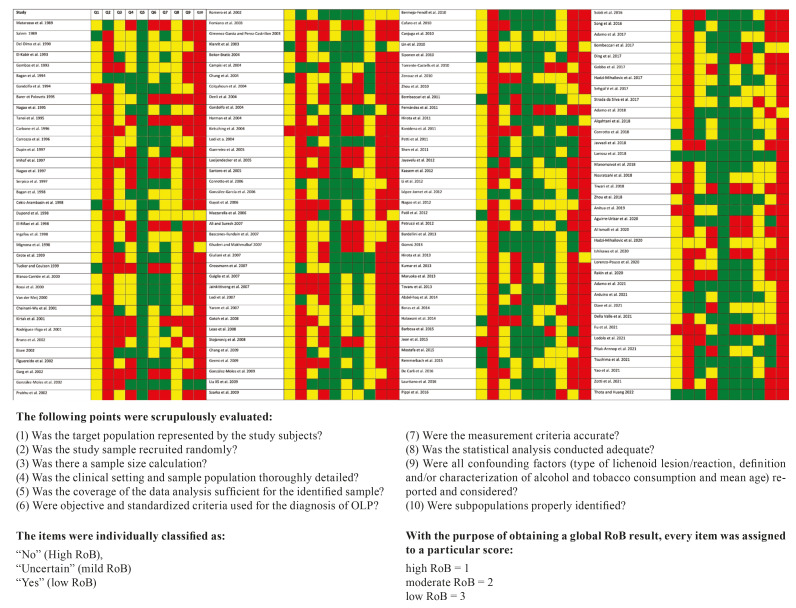
Quality Plot depicting the risk of bias in individual studies, critically appraising ten domains, using a method specifically designed for systematic reviews addressing questions of prevalence (developed by Joanna Briggs Institute, University of Adelaide, South Australia).

According to our qualitative analysis, Fig. 1 (Quality plot) depicts results following our risk of bias (RoB) analysis. As seen in our recent systematic reviews and meta-analyses published (3,4), higher results of risk of bias are related to items Q2, Q9 and Q10.

- Quantitative evaluation

Table 2 summarizes the pooled results obtained in meta-analyses, more detailed displayed in the supplementary information (Supplements 4, 5, 7, 10, 13, 15, 17, 18, 21, 22, 24, 25, 28, 29, 32, 33, 35, 36).

**Table 1:**
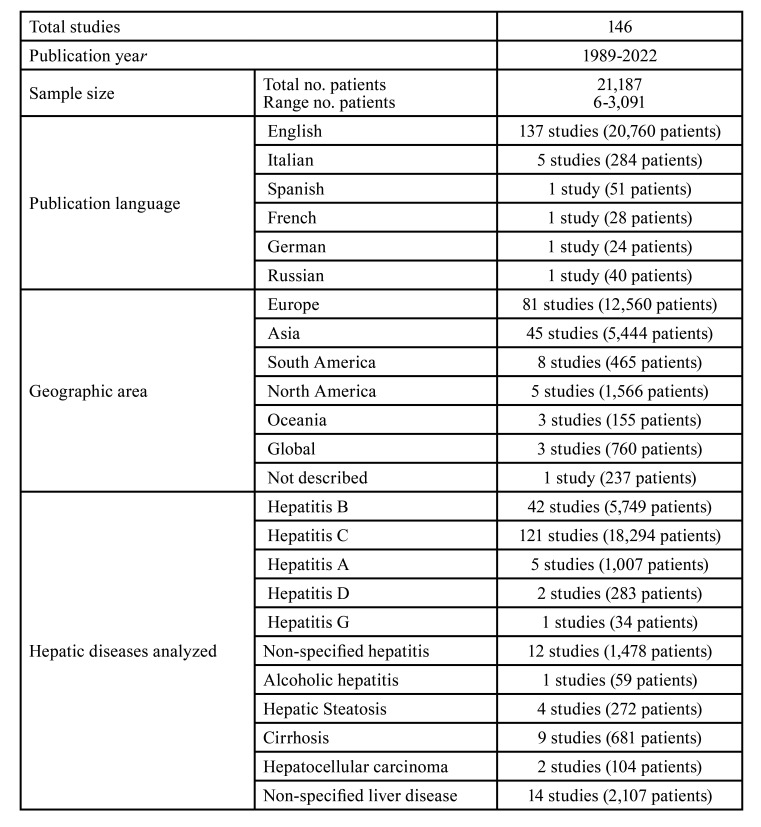
Characteristics of the studies included in the meta-analysis.

**Table 2:**
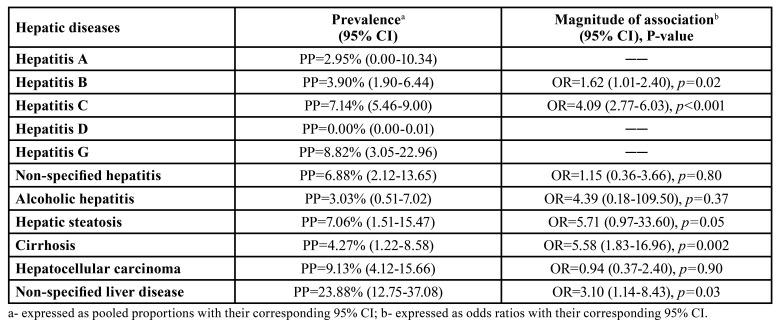
Prevalence and magnitude of association of hepatic diseases in patients with OLP.

**Figure 2:**
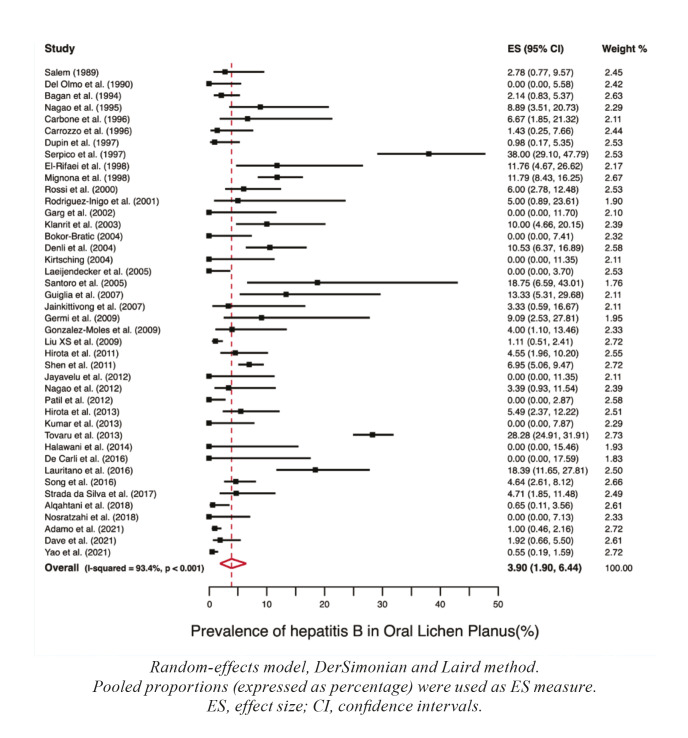
Forest plot graphically representing the meta-analysis of the prevalence of Hepatitis B in patients with OLP.

**Figure 3:**
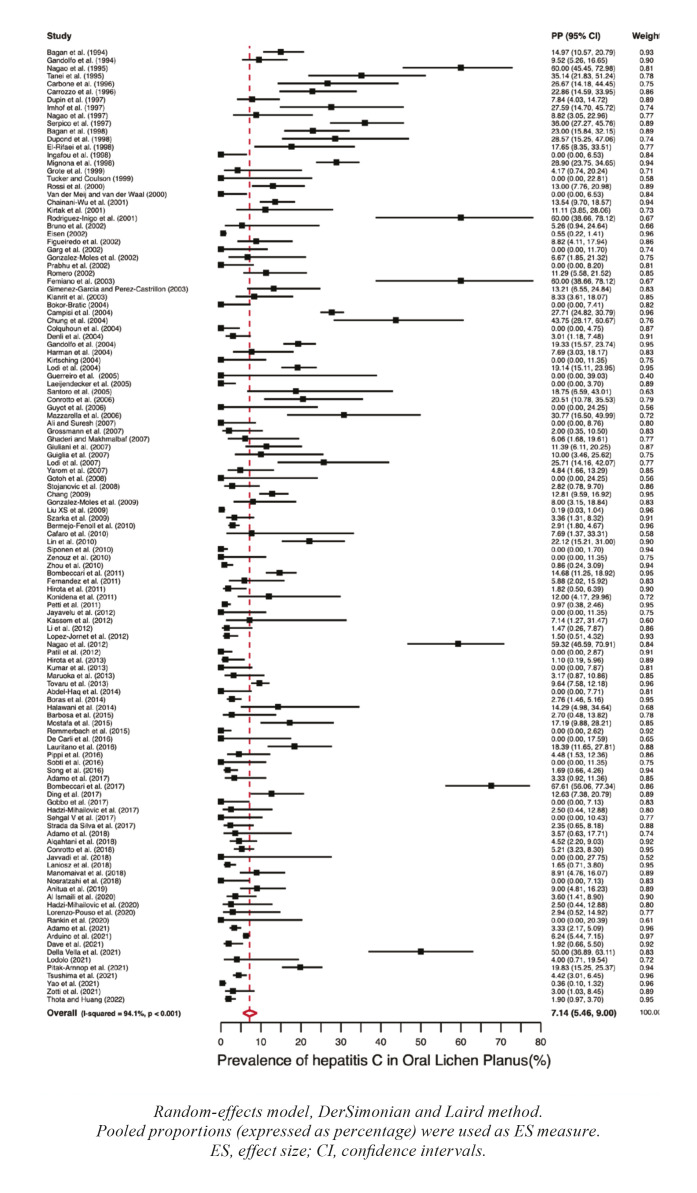
Forest plot graphically representing the meta-analysis of the prevalence of Hepatitis C in patients with OLP.

**Figure 4:**
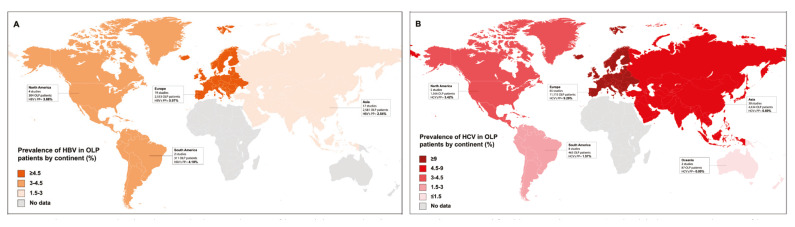
These maps depict the pooled prevalence of hepatitis B and C in OLP patients stratified by continents. A) The highest prevalence of hepatitis B was found in Europe (PP=5.97%), while the lowest was found in Asian countries (PP=2.54%); B) Europe also represented the continent with the highest prevalence of hepatitis C (PP= 9.29%), while no cases were detected in Oceania (PP=0.00%).

Prevalence : Meta-analyses for prevalences of hepatic diseases in patients suffering from OLP were calculated, finding the following results: hepatitis A (PP=2.95%, 95%CI=0.00-10.34), hepatitis B (PP=3.90%, 95%CI=1.90-6.44), hepatitis C (PP=7.14%, 95%CI=5.46-9.00), hepatitis D (PP=0.00%, 95%CI=0.00-0.01), hepatitis G (PP=8.82%, 95%CI=3.05-22.96), non-specified hepatitis (PP=6.88%, 95%CI=2.12-13.65), alcoholic hepatitis (PP=3.03%, 95%CI=0.51-7.02), hepatic steatosis (PP=7.06%, 95%CI=1.51-15.47), cirrhosis (PP=4.27%, 95%CI=1.22-8.58), hepatocellular carcinoma (PP=9.13%, 95%CI=4.12-15.66) and non-specified liver disease (PP=23.88%, 95%CI=12.75-37.08). Forest plots representing the prevalences of hepatitis B and C in patients with OLP are found in Fig. 2, Fig. 3 respectively, while Fig. 4 depicts these prevalences by continents.

Magnitude of association: Hepatic diseases were significantly more frequent in patients with OLP than in general population for hepatitis B (OR=1.62, 95%CI=1.01-2.40, *p*=0.02), hepatitis C (OR=4.09, 95%CI=2.77-6.03, *p*<0.001), hepatic steatosis (OR=5.71, 95%CI=0.97-33.60, *p*=0.05), cirrhosis (OR=5.58, 95%CI=1.83-16.96, *p*=0.002), non-specified liver disease (OR=3.10, 95%CI=1.14-8.43, *p*=0.03). The rest of variables showed no significant associations (*p*>0.05).

- Quantitative evaluation (secondary analyses)

All results of secondary analyses can be found in the supplementary information (Supplements 4, 6, 8, 9, 11, 12, 14, 16, 19, 20, 23, 26, 27, 30, 31, 34, 37-45).

## Discussion

The results of our systematic review and meta-analysis suggest the existence of a significantly higher prevalence of hepatic diseases (HB, HC, liver cirrhosis and hepatic steatosis) that behave as risk factors for the development of hepatocellular carcinoma in patients with OLP. Patients with OLP have a prevalence of HB of 3.90%, being 1.62 times more prevalent compared to patients without OLP (*p*=0.02). The risk of developing HB in OLP shows significant geographical differences (*p*=0.03), being Europe the continent with the highest prevalence (5.97% of European patients with OLP have HB). These results derive from the analysis of 42 primary-level studies that recruited 5,749 patients with OLP. HBV is considered a prominent hepatic oncovirus responsible for approximately 60% of hepatocellular carcinomas in Africa and Asia, and for 20% of these tumors in Western countries (16). HBV integrates its DNA into the host cell genome inducing insertional mutagenesis with activation of oncogenes (17). The site where HBV DNA is most frequently integrated is the TERT promoter, which induces telomerase overexpression -an enzyme responsible for telomere length maintenance- whose overregulated activity protects cells from senescence and promotes cell transformation (18). This oncogenic mechanism is considered to be the most frequent, although not the only one, of those developed by HBV.

Patients with OLP also present a high prevalence of HC (7.14% of patients with OLP suffer HC). The risk of developing HC is 4.09 times higher in OLP compared to healthy population (*p*<0.001). This result was obtained from the analysis of 121 primary level studies that collected 18,294 patients with OLP. As in HB, the risk of developing HC in OLP shows significant geographical differences (*p*<0.001), with Europe also being the continent with the highest prevalence (9.29% of European patients with OLP associate HC). HC is the most common underlying liver disease in patients with hepatocellular carcinoma in North America, Europe and Japan (10). HCV is an RNA virus that consequently does not integrate its genome and therefore does not drive an oncogenic effect mediated by insertional mutations; instead, the risk of hepatocellular carcinoma linked to chronic HCV infection is related to the cirrhosis associated to virus infection and to the oxidative stress caused by chronic inflammation. It should be noted that with direct-acting antiviral therapy, which achieves a sustained virological response in a high percentage of patients, a 50-80% reduction in the risk of developing hepatocellular carcinoma is obtained (19).

Patients with OLP also have a high prevalence of hepatic steatosis (7.06% of patients with OLP), with a risk of developing this hepatic disorder 5.71 times higher in OLP vs healthy population (*p*=0.05). Although this result is derived from only 4 studies and 272 patients with OLP, a remarkable magnitude of association is observed that increases the quality of evidence in this regard (20). Both alcoholic and nonalcoholic hepatic steatosis behave as risk factors for liver cirrhosis and, therefore, for hepatocellular carcinoma. In addition, nonalcoholic hepatic steatosis is recognized as the precursor for hepatocellular carcinoma in patients with type 2 diabetes and obesity (10). Non-alcoholic hepatic steatosis, secondary to the increase of obesity and type 2 diabetes prevalence, has become the most common cause of liver cirrhosis in many areas of the world, and is responsible for 15-20% of hepatocellular carcinoma cases in Western countries (21). It should also be noted that 25-30% of hepatocellular carcinoma cases associated with non-alcoholic hepatic steatosis occur in the absence of cirrhosis (10).

OLP is also associated with a high prevalence of liver cirrhosis (4.27% of patients with OLP suffer from cirrhosis). The risk of cirrhosis in OLP is 5.58 times higher than in the healthy population (*p*=0.002). It is reasonable to hypothesize that this risk derives essentially from the high prevalence of diseases predisposing to the development of cirrhosis in patients with OLP (HB, HC and hepatic steatosis). The essential changes that occur in cirrhosis promote endothelial cell migration, neoangiogenesis and fibrosis (22). In cirrhotic tissue, senescent hepatocytes release chemokines that interfere with senescence-mediated antitumor surveillance and impair immune-mediated tumor suppression (23). Since the histological substrate in cirrhosis is widely spread throughout the hepatic tissue, a permissive microenvironment for tumor development is present, which is referred to as the field cancerization effect. In a percentage of patients with hepatic fields of cancerization, an overregulation of TGFβ signaling, T-cell depletion and overexpression of immune checkpoints have been demonstrated, which was associated with an increased risk of hepatocellular carcinoma (24).

The results of our study show that patients with OLP have a prevalence of hepatocellular carcinoma of 9.13% of cases. Although this prevalence is obtained from the analysis of two studies and the results are not very robust, the lower limit of the confidence interval (4.12-15.66) point out that the prevalence of hepatocellular carcinoma in OLP is higher than 4.12%, which is notably higher than the prevalence of hepatocellular carcinoma in the general population (0.07%)(25). Further research is needed in this aspect to confirm this important association. In this regard, it is also worth noting that HCV infection also behaves as a risk factor for the development of oral cancer in patients with OLP, as reported by our research group (26). The reasons for this association are unknown, although a mechanism linked to the immune response that HCV triggers in the oral mucosa could be hypothesised.

According to our tool Joanna Briggs Institute all studies were not methodologically designed and conducted with the same rigor. However, our meta-analysis shows that the risk of bias does not behave significantly as a source of heterogeneity, not influencing the variation of the distributions of the prevalences of the investigated liver pathologies in patients with OLP. Nevertheless, we believe that the studies should be well designed and future studies could follow our recommendations to standardize future research.

Some limitations are also identified in our present systematic review and meta-analysis. Firstly, a substantial degree of expected inter-study heterogeneity is found in some of our results in the meta-analyses. On this basis, we used random-effects in the statistical analyses and performed subgroup analyses in an attempt to identify possible sources of heterogeneity (meta-analyses by geographic area and sex). Meta-regression analyses were also carried out, estimating the proportion of the inter-study variance explained by covariates. Furthermore, two potential sources of clinical and methodological heterogeneity could be related with strategies utilized in the different studies for the diagnosis of the hepatic diseases, and with the chronicity of these diseases. Secondly, the lack of data provided by most of screened studies did not allow us to obtain a large number of observations in the secondary analyses. More precise datasets should be reported in future investigations in this context. Finally, most of the systematically reviewed primary-level studies were of retrospective nature. Therefore, another important recommendation of the present work is the development of better designed studies in the future, preferably prospective cohorts. Notwithstanding the limitations, our study provides originality, being the first systematic review covering the spectrum of hepatic diseases in OLP. The variables we have studied are of importance in clinical practice, as patients with OLP could be referred to specialists for the assessment and treatment of hepatic comorbidities. Furthermore, the thoroughness of our search strategy allowed us to find a wide range of potentially eligible studies of which 146 studies (21,187 patients) were included.

## Conclusions

Our results demonstrate that patients with OLP are predisposed to develop hepatic diseases that behave as risk factors for hepatocellular carcinoma, such as hepatitis B, hepatitis C, hepatic steatosis, and liver cirrhosis. It is also likely, also and in relation to our results, that patients with OLP are at an increased risk of developing hepatocellular carcinoma. It should be taken into consideration that some of these disorders may not show symptoms for some time and therefore go unnoticed. Consequently, and as a conclusion, it seems reasonable to suggest that patients with OLP could be investigated to rule out the presence of hepatic disease (hepatitis serology and ultrasound studies), which can lead to hepatocellular carcinoma, allowing an early diagnosis that would help to a better approach to liver disease and a noTable improvement in prognosis in terms of both progression and severity.
